# Waist-to-height ratio and skipping breakfast are predictive factors for high blood pressure in adolescents

**DOI:** 10.1038/s41598-020-73355-y

**Published:** 2020-10-07

**Authors:** C. Aparicio-Cercós, M. Alacreu, L. Salar, L. Moreno Royo

**Affiliations:** 1 Community Pharmacy, SEFAC, Valencia, Spain; 2 Embedded Systems and Artificial Intelligence Group, Universidad Cardenal Herrera-CEU, CEU Universities, Alfara del Patriarca, Valencia, Spain; 3 Department of Pharmacy, Universidad Cardenal Herrera-CEU, CEU Universities, C/Ramón y Cajal s/n, Alfara del Patriarca, 46115 Valencia, Spain

**Keywords:** Risk factors, Health services

## Abstract

The purpose of this study was to estimate the prevalence of high blood pressure (HBP) in adolescents of the Valencian Autonomous Community (VC) in Spain. Besides, its association with other risk factors related to cardiovascular disease (CVD) or arterial hypertension (AHT) in order to increase our knowledge of public health and to provide advice about healthy diets**.** We conducted a multicentre, observational, cross-sectional, epidemiological study in a sample of 4402 adolescents from 15 schools during the 2015–2016 school year. The participants were aged between 11 and 18 years, and any individuals already diagnosed with AHT were excluded. In addition to the *Physical Activity Questionnaire for Adolescents* (PAQ-A), *Evaluation of the Mediterranean Diet Quality Index* (KIDMED), a lifestyle habits survey, the waist-to-height ratio (WtHR), and body mass index (BMI) were calculated for each participant. Informed Consent was obtained from Parents of the adolescents involved in the current study. The study received approval from the University ethics committee and all procedures were conducted in accordance with the tenets of the Declaration of Helsinki. Chi-squared, Student t-tests, and ANOVA statistical analyses showed that 653 (14.8%) adolescents had previously undiagnosed HBP and that was significantly associated with male sex (*p* < 0.001), age over 15 years (*p* < 0.05), and height, weight, waist circumference, WtHR, BMI, and skipping breakfast. Based on the data we obtained in this study, the modifiable factors that influence HBP in adolescents were WtHR, BMI, and skipping breakfast.

## Introduction

Cardiovascular diseases (CVD) are currently the leading cause of death in the world with more people dying every year from a pathology related to cardiovascular health than from any other cause. The World Health Organization (WHO) estimated that in 2016, 15.2 million people died from CVD; this represents 27% of all deaths recorded around the world and was the leading cause of death for the past 15 years^1^. In the United States this rate reached 34%^2^, while in the European Union, this figure was 40%^3^.

According to the recent report, *Deaths broken down by cause*, prepared by the Spanish National Institute of Statistics (INE in its Spanish initialism of Instituto Nacional de Estadística), CVD was below the European average but was still the leading cause of death in Spain, accounting for 28.8% of all deaths and even exceeding deaths by cancer or respiratory diseases. The Valencian Autonomous Community (VC) placed tenth with respect to all the other communities in Spain, with a gross mortality rate of 274 per 100,000 inhabitants^4^.

The European Union (EU) Science Centre reported that production losses due to mortality and morbidity associated with CVDs cost the EU 54 billion euros in 2015 and the total cost of providing health care (including medical care, medications, and lost productivity) to people with CVD was 45 billion euros^5^, Spain accounted for 2% of that cost^6^. By 2020, this figure is expected to reach 122.6 billion euros in Spain and approximately 178.9 billion euros in the United States^7^.

The factors that influence CVD can be grouped into genetic and biological factors (e.g., hypertension, dyslipidaemia, or diabetes) whose negative influence is modulated by behavioural factors such as diet, physical activity, and toxic habits (e.g., tobacco or alcohol use), which in turn depend on structural factors including socioeconomic and education levels and characteristics of society itself^8^. In addition to inducing CVD, these risk factors are causally related to other non-communicable diseases such as cancer, diabetes mellitus, or chronic obstructive pulmonary disease^9^.

Arterial hypertension (AHT) is one of the arteriosclerotic disease risk factors that can be modified from an early age. In children and adolescents, it remains a major problem and is a prominent public health concern. The latest epidemiological studies reveal that the prevalence of paediatric AHT varies from 3% in the general population to 25% in obese children^10^. Previous studies have shown that high blood pressure (HBP) in childhood is the strongest predictor of AHT in adults^11^. A cut-off age of 16 years is established by the European Societies of Hypertension Guides for the management of AHT in children and adolescents (prepared in 2016 based on that of 2009), after which the reference figures for diagnosis in adults are followed^12^.

The prevalence of hypertension in childhood increases with obesity which itself is a potential problem because obesity is associated with the appearance of comorbidities in childhood and its persistence into adulthood increases the risk of CVD^13^. The Aladino Study conducted in Spain in 2015^14^ indicated that, based on WHO growth standards, 23.2% of Spanish adolescents were overweight (22.4% male and 23.9% female) and the prevalence of obesity was 18.1% (20.4% male and 15.8% female).

In addition, it has been shown that during childhood and adolescence, waist circumference (WC) significantly correlates with the body mass index (BMI) and percentage of body fat. WC is considered a good indicator of obesity in children and adolescents but is age and sex dependent and so is expressed as corresponding percentiles. In contrast, the ratio between the WC and height, also called the waist-to-height ratio (WtHR), remains stable during growth and so does not need to be compared with age percentiles^15^. WtHR has a uniform behaviour in correlation with age, against the increasing linear behaviour of the BMI, which it is also has a simpler mathematical calculation^16^. The habit of eating breakfast also appears to be related to preventing obesity because adolescents who regularly consume breakfast have a lower body fat content^17^.

Moreover, adolescents are increasingly using new technologies as their main form of communication and as a source of social and academic support. Many hours can be spent using such devices even though they have been associated with poor sleep^18^ and a considerable increase in physical inactivity^19^. Furthermore, despite their known association with venous and arterial thrombi^20^ and direct relationship with HBP over time^21^, the use of contraceptives (either to prevent pregnancy or to improve dysmenorrhea or menorrhagia^22^) has become increasingly common among adolescent girls.

Adolescence is a stage of considerable risks which can be determined by the social environment^23^. Thus, it is important to try to influence risk factors such as adherence to a healthy diet, physical activity, tobacco and alcohol consumption at this stage because these are much harder to modify during adulthood. In this present study we aimed to determine which of these modifiable risk factors should be targeted from early ages.

Our main objective was to estimate the prevalence of HBP in adolescents from the VC, and to investigate its association in this population with other risk factors related to CVD and AHT such as obesity and its possible causes including diet quality, physical inactivity, exposure to sedentary habits. We used these data to create an adjusted model to estimate the probability of adolescents suffering from HBP based on the factors that most strongly influenced this outcome.

## Materials and methods

### Sample population

We planned an observational, epidemiological, cross-sectional, multicentre study, with a **target population** of 482,143 adolescents. Calculated by consultation in the Spanish National Institute of Statistics database, filtering by community (selection: Valencia Community), age group (selection: 10–14 and 15–19 years) and year of consultation (selection: 2015), independently of sex and place of origin (Spanish or foreigners)^24^. We established the **accessible population** as the 346,488 students enrolled in compulsory secondary education (ESO in its Spanish initialism of Enseñanza Secundaria Obligatoria), Baccalaureate, or vocational training centres in the 3 provinces comprising the Valencia Community.

The academic directors from 21 of these centres where informed about this study by telephone and 15 of them indicated their willingness to participate in it (2 in Castellon, 2 in Alicante, and 11 in Valencia). At a meeting with each director, the project and the planned measures for informing the students were explained and they were provided with the informed consent form (Annex 1) they should give them to the guardians of the student target population. We also agreed the day on which the study would be carried out and therefore when the completed and informed consent forms signed would be required. A signature from a guardian was required even for students of legal age at the time of the study.

The field work was carried out from October 2015 to May 2016. Prior to commencing the study, a group of 33 community pharmacists, volunteers, and Spanish Society of Family and Community Pharmacy (SEFAC) partners, were trained in the data collection requirements so that the data from each centre would be standardised.

A total of 4443 adolescents participated; 41 of these did not meet the inclusion criteria: age 11–18 years (7 cases), provision of a signed informed consent (7 cases), diagnosis with HBP (20 cases), and provision of all the requested information (7 cases). Thus, the final sample size was 4402 adolescents, which quadrupled the sample size required of 1065 adolescents. Data based on the classic statistical formula for estimating the necessary sample size to estimate the prevalence of HBP in this group, based on a 50% prevalence rate (the least favourable statistical possibility), with an accuracy of 3% and a 95% confidence level.

### Ethical considerations

This research was part of the SEFAC *Mepafac Study*^25^ (translated from Spanish as ‘measurement of blood pressure and health education in cardiovascular risk factors in schools by community pharmacists’) and was approved by the Research Ethics Committee at the CEU San Pablo University on 28 July 28 2014 (code №: 084-14).

Informed Consent was obtained from Parents of the adolescents involved in the current study. The study received approval from the University ethics committee and all procedures were conducted in accordance with the tenets of the Declaration of Helsinki.

### Survey variables

We designed a wide-ranging anonymous survey to collect data from adolescents, which included items on different topics.

#### Sociodemographic, anthropometric, and health variables

- Province: Castellon, Valencia, and Alicante.
- Academic level: 1st, 2nd, 3rd, and 4th year of the ESO; 1st and 2nd year of the Baccalaureate; and 1st and 2nd year of vocational training.
- Sex: female (F) and male (M).
- Age: in years.
- Height: in centimetres; recorded by collaborating pharmacists using a calibrated portable stadiometer.
- Weight: in kilograms; recorded by collaborating pharmacists using a calibrated scale.
- WC: in centimetres around the relaxed abdomen, above the upper edge of the iliac crest^39^; recorded by collaborating pharmacists using a flexible measuring tape.
- Hours of sleep: As daily, number of hours of sleep.
- Diagnosis of diseases: HBP, diabetes, high cholesterol, heart-related diseases, kidney-related diseases, others.
- Chronic medications: yes or no.
- Frequency of menstrual cycle regulators: never, daily, weekly, and monthly.

Additional classification variables were calculated based on the above:

- WtHR: WC/Height.
- Categorised WtHR: normal weight (WtHR ≤ 0.5) and risk of being overweight (WtHR > 0.5), according to established standards^26^.
- BMI: Weight/Height^2^, expressed as kg/m^2^.
- Categorised BMI: Underweight, normal weight, overweight, and obese as defined by Orbegozo^27^ (with cross-sectional study tables) according to sex and age (BMI < P3, BMI < 85 P85, BMI ≥ P85, BMI ≥ P95, respectively).

#### Variables related to blood pressure

- SBP: systolic blood pressure, expressed in mmHg.
- DBP: diastolic blood pressure, expressed in mmHg.

Both these variables are the means of repeated measurements obtained by the collaborating pharmacists using validated and calibrated Omron M3 (HEM-7200-E) oscillometric sphygmomanometers, with the following protocol: after 5 min at rest, two measurements of both the SBP and DBP were recorded, leaving 3 min between each measurement. If the difference between these exceeded 5 mmHg, a third measurement was recorded. If a difference exceeding 5 mmHg was still noted, a fourth measurement was recorded. Two cuff sizes were used: S (18–22 cm) and M (22–32 cm) according to the circumference of the youngsters' arms.

- Family clinical history of AHT: yes or no.
- Family member with AHT: father, mother, grandfather, and/or sibling.

Additional blood pressure classification variables were calculated based on the above:

- Blood pressure category: normal, normal-elevated, grade 1 hypertension, grade 2 hypertension, grade 3 hypertension, isolated systolic hypertension; according to the classification in the European Hypertension Societies Guide for adolescents aged under 15 years (prepared in 2016). From the age of 16 years, we followed the reference criteria for diagnoses in adults^12^ (Table 1).
- Type of HBP: classification of adolescents into 4 groups: (1) normal SBP and DBP; (2) high SBP only; (3) high DBP only; and (4) high SBP and DBP.
- Adolescents were classified into two groups: HBP (one or both SBP and DBP high) and normal BP.

**Table 1:** Classification for the diagnosis of hypertension based on the percentile distribution of age, sex, and height for children aged under 16 years as well as the classification for patients aged over 16 years.

Category	0–15 years SBP and/or DBP percentile	Older than 16 years SBP and/or DBP values (mmHg)
Normal	< P90	< 130/85
Normal-elevated	≥ P90 to < P95	130–139/85–89
GRADE 1 hypertension	P95–P99 + 5 mmHg	140–159/90–99
GRADE 2 hypertension	> P99 + 5 mmHg	160–179/100–109
GRADE 3 hypertension		≥ 180/ ≥ 110
Isolated systolic hypertension	SBP ≥ P95 and DBP < P90	≥ 140/ < 90

*P* percentile.

#### Physical activity

- The *Physical Activity Questionnaire for Adolescents* (PAQ-A; Annex 2), comprises 9 questions that evaluate the physical activity performed by adolescents in the 7 days prior. The final score is calculated using the first 8 questions; the arithmetic mean is obtained from question 1, which is averaged with the score of the following 7 questions. The final test result is a score ranging from 1 to 5.
- Categorised PAQ-A classifies adolescents into three groups according to their score for their engagement in physical activity performed in the 7 days prior: low (PAQ-A < 2), moderate (2 ≤ PAQ-A ≤ 3), or high (PAQ-A > 3)^28^.

#### Dietary habits

- *Evaluation of the Mediterranean Diet Quality Index* (KIDMED) questionnaire (Annex 3), comprising 16 questions, some with positive connotations when answering YES (with a score of + 1 point) and others with negative connotations (with a score of − 1 point; questions 6, 12, 14, and 16).
- Categorised KIDMED: this classifies adolescents into three groups according to their adherence to the Mediterranean diet: poor-quality diet (KIDMED ≤ 3); improvable (3 < KIDMED < 8); and optimal (KIDMED ≥ 8)^29,30^.
- Consumption frequency of candies/sweets: never, sometimes, and daily.
- Consumption frequency of pastries: never, sometimes, and daily.
- Consumption frequency of nuts: never, sometimes, and daily.
- Consumption frequency of snacks: never, sometimes, and daily.
- Consumption frequency of canned foods: never, sometimes, and daily.
- Consumption frequency of burgers: never, sometimes, and daily.
- Consumption frequency of sauces: never, sometimes, and daily.
- Consumption frequency of muscle-gain supplements: never, sometimes, and daily.
- Consumption frequency of weight-loss products: never, sometimes, and daily.

#### Toxic habits

- Frequency of tobacco use: never, sometimes, and daily.
- Frequency of alcohol consumption: never, sometimes, and daily.

#### Technology-use habits

- Television: time spent per day watching television (in hours).
- Videogames console use: time spent per day using a videogames console (in hours).
- Computer: time spent per day using a personal computer (in hours).
- Smartphone: time spent per day using a smartphone (in hours).

The measurements recorded by the pharmacists (height, weight, WC, SBP, and DBP) were carried out while the adolescents were wearing light clothes and without shoes, in a private area sectioned off specifically for this work.

### Statistical analyses

The adolescents anonymously answered the survey on an ad hoc record sheet and then the pharmacists completed it with their measurements. A Microsoft Access form was designed specifically for the purpose of the digitalisation and storage of the surveys by each collaborator. Once complete, each database was exported to a Microsoft Excel spreadsheet, and finally, all these were combined to obtain the definitive database.

Statistical analysis of the data was performed using R advanced statistical software. The prevalence of HBP was estimated with 95% confidence. The association between HBP and the qualitative variables was tested by comparing percentages using χ^2^ and Fisher exact tests, and with the quantitative variables using Student *t*, Wilcoxon, ANOVA, and Kruskal–Wallis tests. Fisher's exact test was used when some contingency table cells have a very low sample size with respect to the global total.

A logistical model was adjusted to estimate the probability of HBP according to patient sex and WtHR. We also assessed the association between WtHR or categorised WtHR with modifiable habit factors using Pearson, χ^2^, and Fisher exact tests. For statistical inferences the *p*-values of < 0.05 were considered statistically significant.

## Results

We recruited 4402 adolescents aged between 11 and 18 years to this study; none had had a prior alert about their blood pressure, but 653 of them (14.8%) presented HBP. Thus, we estimated that 13.8% to 15.9% of adolescents in the VC have HBP.

**Figure 1:**
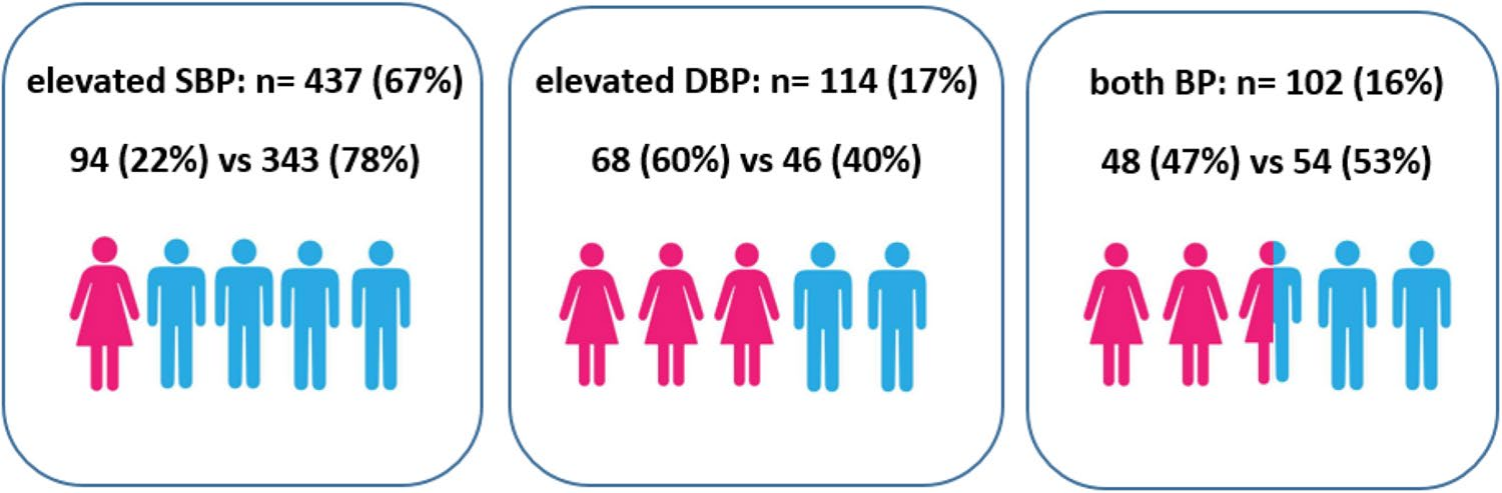
Distribution of different types of HBP in adolescents according to sex.

As shown in Fig. 1, there are three main HBP presentations which have different profiles with respect to sex: elevated systolic blood pressure (SBP), elevated diastolic blood pressure (DBP), or simultaneously elevated systolic and diastolic blood pressure; these scenarios presented in 437 (66.9%), 114 (17.5%), and 102 (15.6%) adolescents, respectively.

We tried to correlate HBP (regardless of the presentation) with the sociodemographic and anthropometric metrics as well as physical activity, dietary, toxic, and technology-use habits of the study cohort. Table 2 shows these data broken down as non-modifiable factors (sex, age, and family history of AHT) and modifiable factors (WtHR, BMI, PAQ-A, and KIDMED as categorised variables, and consumption of weight-loss products, tobacco, or alcohol).

**Table 2:** Association between HBP presentation and sex, age, family history of HBP, WtHR, BMI, PAQ-A, or KIDMED as categorised variables, or a daily breakfast-eating habit, or consumption of weight-loss products, tobacco, or alcohol.

	High BP *n* (%)	Normal BP *n* (%)	Total *n* (%)	*p*-value
**Non-modifiable factors**				
Sex				
Male	443 (19.9%)	1782 (80.1%)	2225 (100%)	< 0.001^a^
Female	210 (9.6%)	1967 (90.4%)	2177 (100%)	
Age				
11 years	7 (13.9%)	45 (86.5%)	52 (100%)	0.0300^b^
12 years	101 (13.9%)	627 (86.1%)	728 (100%)	
13 years	118 (14.2%)	712 (85.8%)	830 (100%)	
14 years	124 (15.1%)	699 (84.9%)	823 (100%)	
15 years	132 (17.0%)	644 (83.0%)	776 (100%)	
16 years	72 (11.2%)	571 (88.8%)	643 (100%)	
17 years	80 (17.5%)	378 (82.5%)	458 (100%)	
18 years	19 (20.7%)	73 (79.3%)	92 (100%)	
Family clinical history of AHT				
Yes	141 (15.6%)	762 (84.4%)	903 (100%)	0.4593^a^
No	512 (14.6%)	2987 (85.4%)	3499 (100%)	
**Modifiable factors**				
Categorised WtHR				
Normal weight	477 (12.5%)	3341 (87.5%)	3818 (100%)	< 0.001^a^
Overweight	176 (30.1%)	408 (69.9%)	584 (100%)	
Categorised BMI				
Underweight	2 (3.6%)	54 (96.4%)	56 (100%)	< 0.001^b^
Normal weight	377 (11.2%)	3000 (88.8%)	3377 (100%)	
Overweight	123 (24.4%)	382 (75.6%)	505 (100%)	
Obese	151 (32.5%)	313 (67.5%)	464 (100%)	
Categorised PAQ-A				
Low activity levels	206 (13.0%)	1374 (87.0%)	1580 (100%)	0.0199^a^
Normal activity levels	364 (16.3%)	1874 (83.7%)	2238 (100%)	
High activity levels	83 (14.2%)	501 (85.8%)	584 (100%)	
Categorised KIDMED				
Optimal diet	150 (14.9%)	855 (85.1%)	1005 (100%)	0.8007^a^
Improvable diet	424 (15.0%)	2405 (85.0%)	2829 (100%)	
Poor-quality diet	79 (13.9%)	489 (86.1%)	568 (100%)	
Breakfast every day				
Yes	511 (14.7%)	2956 (85.3%)	3467 (100%)	0.7322^a^
No	142 (15.2%)	793 (84.8%)	935 (100%)	
Weight-loss products				
Never	641 (15.0%)	3628 (85.0%)	4269 (100%)	0.1425^b^
Sometimes	8 (8.2%)	90 (91.8%)	98 (100%)	
Daily	4 (11.4%)	31 (88.6%)	35 (100%)	
Tobacco				
Never	605 (15.2%)	3365 (84.8%)	3970 (100%)	0.0492^b^
Sometimes	33 (12.2%)	238 (87.8%)	271 (100%)	
Daily	15 (9.3%)	146 (90.7%)	161 (100%)	
Alcohol				
Never	482 (15.5%)	2619 (84.5%)	3101 (100%)	0.1183^b^
Sometimes	169 (13.2%)	1112 (86.8%)	1281 (100%)	
Daily	2 (10.0%)	18 (90.0%)	20 (100%)	
Total	653 (14.8%)	3749 (85.2%)	4402 (100%)	

HBP, high blood pressure; WtHR, waist-to-height ratio; BMI, body mass index; PAQ-A, *Physical Activity Questionnaire for Adolescents;* KIDMED, *Evaluation of the Mediterranean Diet Quality Index.*

Statistical tests: ^a^χ^2^ test; ^b^Fisher exact test.

The prevalence of HBP in male adolescents was double that of their female counterparts (19.9% vs. 9.6%; *p* < 0.05).

The percentage of adolescents with HBP significantly increased after the age of 15 years.

There was no significant association between having a known family history of AHT in a grandparent, parent, or sibling and HBP in these adolescents.

According to the categorised WtHR or BMI, the prevalence of HBP was nearly three times higher among overweight or obese adolescents compared to their normal weight counterparts.

We also observed that the prevalence of HBP was significantly higher among adolescents with normal physical activity levels in the 7 days prior compared to those who had engaged in high levels of physical activity (*p* < 0.05).

There was no statistically significant association between the categorised KIDMED index (which interrogates different aspects of diets), the habit of eating breakfast daily, or the consumption of weight-loss products and HBP among adolescents.

In addition, there was no statistical evidence of any association between HBP and smoking or alcohol consumption in this cohort. However, the SBPs and DBPs are higher in adolescents who consumed alcohol compared to those who abstained from it (mean SBP = 114.03 ± 12.70 mmHg vs. 111.33 ± 12.49 mmHg and mean DBP = 67.52 ± 7.48 mmHg vs. 66.53 ± 7.84 mmHg, respectively; *p* < 0.05). It was striking that, of the adolescents we studied, almost 10% and 30%, respectively, smoked or drank alcohol sometimes or on a daily basis. Both these toxic habits were significantly related to age with a significant increase in tobacco consumption from 16 years and in alcohol consumption from age 15. Interestingly, sex was not associated with tobacco use but was a factor linked to alcohol consumption with significantly more girls than boys reporting that they drank alcohol daily or sometimes (31% vs. 28%, respectively).

According to Student *t*-tests, we detected no significant differences in HBP in association with the daily average time spent viewing television (HBP = 1.57 ± 1.23 h vs. normal BP = 1.65 ± 1.33 h; *p* > 0.05). However, adolescents with HBP spent significantly longer using videogames consoles or personal computers each day than those with a normal BP (0.46 ± 0.95 h vs. 0.39 ± 1.08 h; *p* < 0.05 and 0.85 ± 1.27 h vs. 0.76 ± 1.12 h, respectively; *p* < 0.05). In contrast, daily smartphone use time was significantly shorter among adolescents with an HBP compared to their counterparts with a normal BP (2.67 ± 2.61 h vs. 3.30 ± 3.65 h, respectively; *p* < 0.05).

### Logistical model

To estimate the probability of presenting HBP, we used sex and WtHR as covariates to adjust a logistical model (as summarised in Table 3). These covariates represent two characteristics on the profile of young people. The variable sex is a qualitative characteristic, not modifiable; whose reference category in the model is Female. The WtHR variable is a quantitative, modifiable characteristic. These variables were chosen because they were significantly associated with HBP and their interpretation was relatively simple.

**Table 3:** Logistical model adjustment to sex and WtHR.

Variable	β_i_	*SD*	Wald	d.f.	*p*-value	Exp(β_i_)	95% CI	
							UL	LL
Intercept	− 5.3711	0.3236	− 16.597	1	< 0.001	0.0046	0.0025	0.0087
Sex [male]	0.7639	0.0912	8.376	1	< 0.001	2.1466	1.7975	2.5704
WtHR	7.0831	0.6981	10.146	1	< 0.001	1191.65	304.01	4697.30

β_i_, model coefficients; *SD, s*tandard deviation of the coefficients; d.f., degrees of freedom; Exp(β_i_), odds ratio; UL, upper limit of the 95% confidence interval for the expected odds ratio; LL, lower limit of the 95% confidence interval for the expected odds ratio.

Both BMI and WtHR were significantly associated with HBP and had a strong linear and positive correlation (*p* < 0.001). However, the mean WtHR was more stable than the mean BMI, regardless of age and the blood pressure status of our study participants. Of note, the mean of both indices was higher among adolescents with HBP and were both higher in males than in females. In addition, as a function of age, the mean BMI grew linearly while the behaviour of the mean WtHR was uniform.

**Figure 2:**
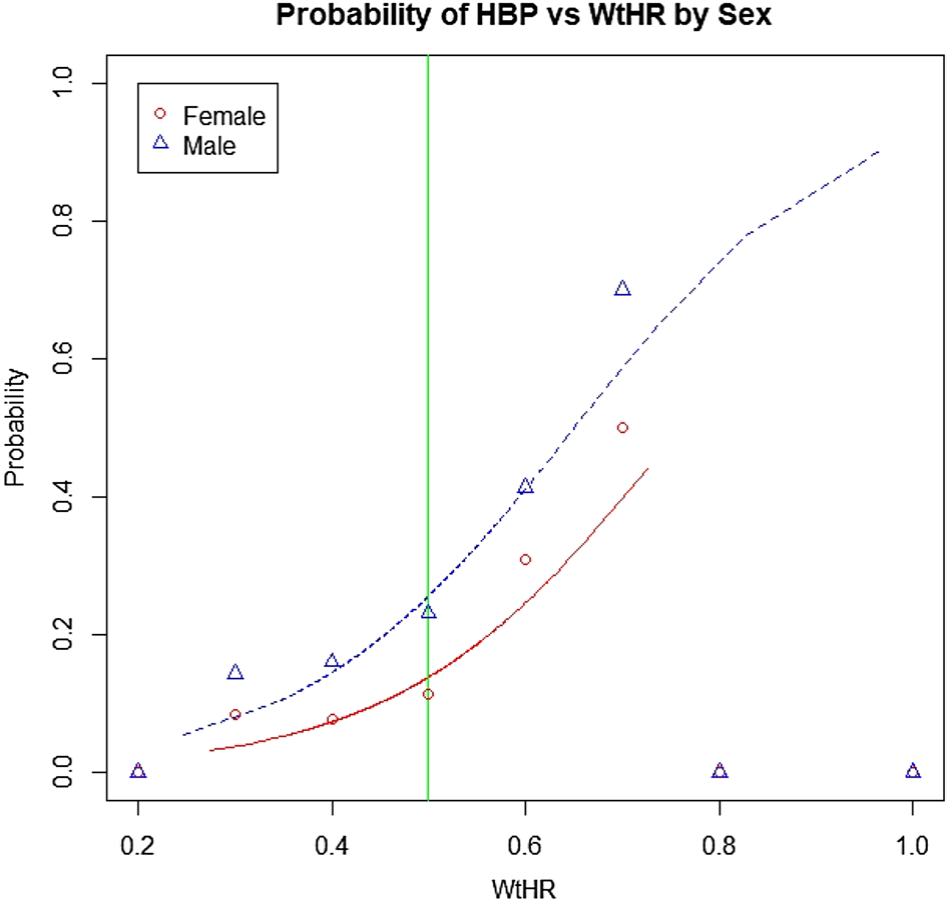
The probability of HBP in each sex as a function of the WtHR. The WtHR = 0.5 line represents the threshold value that separates adolescents with a normal weight from those who were overweight.

Based on this model, the odds of HBP in male adolescents is approximately 1.80 to 2.57-fold higher than for female adolescents with the same WtHRs. Likewise, for adolescents of the same sex, the odds of HBP are expected to increase by 1.77 to 2.33-fold for every 0.1unit increase in WtHR. Figure 2 shows the estimated probabilities of HBP adjusted according to the WtHR and sex. Note that the WtHR = 0.5 line represents a threshold value that separates normal weight adolescents from those who were overweight. The model appears to underestimate the probability of suffering from HBP in either sex for WtHRs lower than 0.8 units.

### Associations with the waist-to-height ratio

Finally, we also analysed the association of modifiable factors with the WtHR, regardless of whether their previous association with HBP in this study had been significant.

As shown in Table 4, although categorised WtHR and BMI were significantly associated, they differently classified 561 adolescents (12.7%) in our sample. Specifically, 88 participants were classified as overweight by the categorised WtHR criteria but as normal weight with the categorised BMI criteria, and 473 adolescents were classified as overweight or obese by the categorised BMI but as a normal weight with the categorised WtHR.

**Table 4:** Association between categorised WtHR and categorised BMI, a daily breakfast-eating habit, and consumption of weight-loss products.

	Categorised WtHR			*p*-value
	Normal weight *n* (%)	Overweight *n* (%)	Total *n* (%)	
**Modifiable factors**				
Categorised BMI				
Underweight	56 (1.5%)	0 (0%)	56 (1.3%)	< 0.001^b^
Normal weight	3289 (86.1%)	88 (15.1%)	3377 (76.7%)	
Overweight	342 (9.0%)	163 (27.9%)	505 (11.5%)	
Obese	131 (3.4%)	333 (57%)	464 (10.5%)	
Breakfast every day				
Yes	3038 (79.6%)	429 (73.5%)	3467 (78.8%)	< 0.001^a^
No	780 (20.4%)	155 (26.5%)	935 (21.3%)	
Weight-loss products				
Never	3728 (98.2%)	541 (96.8%)	4269 (97%)	< 0.001^a^
Sometimes	69 (1.2%)	29 (2.4%)	98 (2.2%)	
Daily	21 (0.6%)	14 (0.8%)	35 (0.8%)	
Total	3818 (100%)	584 (100%)	4402 (100%)	

Statistical tests: ^a^χ^2^ test and ^b^Fisher exact test.

Interestingly, although a daily breakfast-eating habit was not significantly associated with HBP, it did correlate with the categorised WtHR. Specifically, of the adolescents who did not eat breakfast daily, significantly more were overweight.

Significantly, more adolescents who consumed weight-loss products were overweight compared to those that were a normal weight.

**Figure 3:**
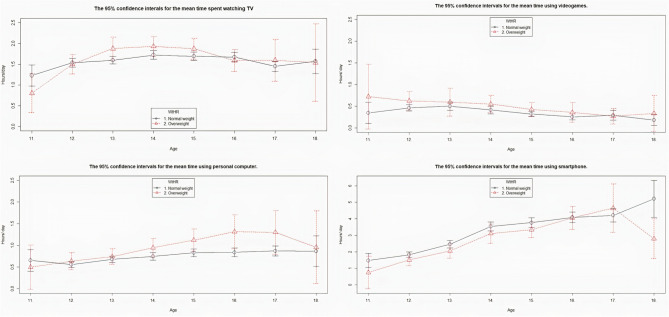
The mean time spent daily by overweight or normal-weight adolescents in different age groups watching television, using videogames consoles, personal computer, or smartphone according to the WtHR criteria. The 95% confidence intervals are shown.

Pearson correlations showed a linear association between WtHR and several habits consistent with a sedentary lifestyle: the daily amount of time spent watching television, using a videogames console, or computer (positive correlation; *p* < 0.05) and smartphone use (negative correlation; *p* < 0.05). Figure 3 shows the mean time normal-weight or overweight adolescents of different age groups would be expected to spend using each of these four technologies according to the WtHR criteria. Note that the time scale used in the first three cases is the same but was extended for smartphone use.

Regardless of age, the mean time spent using technologies was longest for mobile telephones, followed by television viewing, personal computer use, and videogames console use. The average daily time spent watching television, using videogame consoles, or a personal computer was higher among overweight adolescents. However, the opposite was true for smartphone use. Interestingly, the mean time spent watching television was higher among overweight adolescents aged 13 to 15 years. While for overweight adolescents the average time spent using videogames consoles was highest aged 11 years and decreased with age, the mean time spent using a personal computer was highest between 15 and 17 years. In contrast, the average time spent using a smartphone linearly increased with age and was almost always slightly higher among adolescents with a normal weight.

## Discussion

HBP in adolescents is a silent health problem because it is symptomless and difficult to detect. This is because these adolescents do not often go to their family doctor and when they do, their blood pressure is not usually measured. Therefore, the problem of HBP generally goes unnoticed in adolescents and this can increase their risk of developing AHT in adulthood^31^.

This current research indicates that some 13.8% to 15.9% of adolescents have HBP. These figures agree with those published by the American guide for the management of AHT in children and adolescents (15–19% for boys and 7–12% for girls)^2^, although their data significantly differed by race. Similarly, our figures may be subject to variation because of differences in the methods used to measure blood pressure, ethnicities, and cultural and nutritional differences. Some of these differences could also be accounted for by our use of the new American Academy of Paediatrics classification criteria published in 2017^32,33^.

In addition, SBP and DBP are sexually dysmorphic: the mean SBP is significantly higher in boys than in girls while the mean DBP is significantly higher in girls than in boys^12^. Our results also followed this pattern: a high SBP profile was more common in male adolescents than in females (15.3% vs. 4.4%, respectively) but, on the contrary, a high DBP profile was more common among adolescent females than males (3.1% vs. 2.1%, respectively). In addition, in agreement with the published literature^34^, significantly more adolescent males had HBP compared to female adolescents.

At around 14–15 years, the provision of paediatric medical care usually changes over to that of general medicine. Moreover, the criteria for classifying HBP also change from percentiles to absolute values after 15 years of age. Thus, perhaps this change in criteria, together with the hormonal changes experienced by adolescents at around this age, can explain why significantly more adolescents are diagnosed with HBP from this age^12^.

Even though a link between AHT in adulthood and a hereditary genetic component is well established in the scientific literature, we found no relationship between HBP during adolescence and a family clinical history of AHT. Perhaps, although patients with a family history of AHT have a higher risk of suffering from HBP, this problem may not have yet manifested in adolescents^35^.

In agreement with other studies^36^, our results indicate that HBP is associated with being overweight according to both the WtHR and BMI criteria. One meta-analysis found a moderate, positive correlation between SBP and DBP and BMI and WC, as well as a decrease in SBP and DBP by 4.4 mmHg and 3.6 mmHg, respectively, in association with a weight loss of 5.1 kg. Therefore, it seems that reducing body weight to a BMI of < 25 is important in the control of blood pressure^37,38^.

Recommendations of the Spanish Society for the Study of Obesity (SEEDO) have been followed of WC measurement method, above the upper edge of iliac crests (at the level of the navel)^39^. In contrast to many countries, WC normative systems based on WHO/IDF recommendations (midway between the lowest ribs and the iliac crest) have been developed^40^.

Another finding that was compatible with other studies^41^, was the 12.7% discrepancy in the classification of overweight and normal weight individuals when they were classified using categorised WtHR versus categorised BMI. This difference may be because BMI considers the association between height and weight but not the bodily distribution of that weight, meaning that a very heavy athletic body with a narrow WC would tend to be classified as overweight by the BMI but as normal weight by the WtHR. Conversely, a person with a very pronounced and full WC would likely be categorised as overweight by the WtHR but, depending on their height and sex, may be categorised as normal weight by the BMI. Nonetheless, it was more useful to study the WtHR to assess HBP in this current study.

In contrast to recently published results recommending the Mediterranean diet as a means to achieve a normal weight and avoid HBP^38^, we found no significant association between HBP and adherence to this diet, as measured using the KIDMED index. This discrepancy may be because some degree of maturity is required to answer some questions on the KIDMED index, and so we cannot be sure that this questionnaire is appropriate for the youngest participants among our cohort. In a study similar to ours that found a relationship between diet and HBP among younger children, the dietary survey was completed by participants’ guardians^36^. However, another study found no relationship between adherence to a Mediterranean diet and protection from HBP, although this relationship did become significant when the child’s hand grip strength was also taken into account^42^.

Regarding diet, it is important to separately consider the daily habit of eating breakfast. As we also report in this current research, the Helena study^17^ conducted in Europe found that a breakfast-eating habit was related to the lowest body fat percentage and healthiest cardiovascular risk profiles. Likewise, the work carried out by the International Breakfast Research Initiative (IBRI) in Europe and North America, also established the importance of eating breakfast by analysing national and regional food-consumption databases to gain a clearer picture of food and nutrient intake at breakfast^43,44^.

Although smoking is associated with the development of HBP in patients aged over 35 years^45^, our research found no evidence for this association among adolescents. This result is consistent with that obtained in a meta-analysis of 29 studies, which included 192,067 children, and adolescents, which concluded that neither active tobacco consumption nor passive exposure to it were associated with the development of AHT^46^.

The alcohol consumption figures we obtained in this study (29.6%) were similar to those from other studies carried out in Spanish adolescents (28.3%)^47^. We did not find an association between this habit and HBP, however, it is likely that studies with a longitudinal rather than a transversal statistical design will be required to detect any such associations. Nonetheless, there is no doubt that both these toxic habits are harmful, even though it is unlikely that their harmful effects will have yet manifested at such early ages.

Adolescents now spend increasing amounts of time watching television and using other screened devices such as personal computers, videogames consoles, or smartphones. We found that the daily television viewing time was not directly associated with HBP but was higher among overweight adolescents and so this factor was associated with WtHR. However, the time adolescents spent daily using videogames consoles and personal computers was associated both with being overweight and with HBP. Conversely, smartphone use was inversely associated with the same outcomes.

These use habits also evolved with age: younger adolescents watched television and used smartphones for longer, and their use of videogames consoles tended to be replaced by personal computer use, as they got older. Another study found that the time spent watching television or screened devices was indirectly associated with HBP via other risk factors such as obesity, sugary drinks consumption, and inadequate sleep^48^. Conversely, other authors found a direct association between the times spent using such devices and HBP and other parameters that have potentially harmful effects on health^49^.

### Limits

As a self-evaluated survey by young people, it has been difficult for us to consult on some demographic factors such as income, depression, etc. Therefore, these variables have not been investigated. Another limitation is that a proportional sample of adolescents was not obtained in the three provinces but can be considered representative of the accessible population given the sample size achieved.

## Conclusions

HBP is a complex health problem that affects 13.8% to 15.9% of adolescents aged between 11 and 18 years. Compared to their female counterparts, it was twice as prevalent among male adolescents, and was present in 27% of the overweight participants (according to WtHR criteria) in our study. The adjusted logistical model we created allowed us to estimate the probability of HBP based on two covariates.

Some risk factors associated with AHT in adults did not statistically correlate with HBP in adolescents, including a family history of AHT, low physical activity levels, or not following a Mediterranean diet. In addition, some factors were not associated with HBP but did correlate with WtHR; these included a daily habit of eating breakfast and habits consistent with a sedentary lifestyle such as watching television for long periods, and excessive use of videogames consoles, personal computers, and smartphones. Adequate control of these factors, combined with a healthy lifestyle, could contribute to reducing WtHR and indirectly prevent the appearance of HBP.

## Supplementary information

Supplementary Information.

## Data Availability

According to the SEFAC statutes, we are unable to share the original data from this study. For data inquiries, the SEFAC (the database owners) must be contacted.
