# Supplementary material for: Waist-to-height ratio and skipping breakfast are predictive factors for high blood pressure in adolescents
Source: Sci Rep. 2020 Oct 7;10:16704. doi: 10.1038/s41598-020-73355-y (PMC7542155; doi:10.1038/s41598-020-73355-y)
Supplement: Supplementary file 1 — Supplementary Information. [file 41598_2020_73355_MOESM1_ESM.docx]

**ANNEX 1. INFORMED CONSENT**


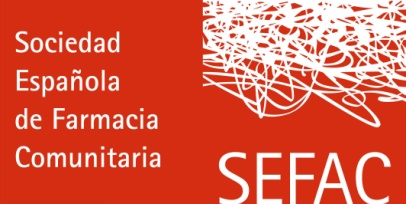


Dear parents/guardians:

Your child’s educational centre has decided to collaborate with the scientific society of community pharmacists, the SEFAC, in its investigative research work, *The measurement of blood pressure in adolescents: education about cardiovascular risk factors.*

In addition to measuring their blood pressure values, each study participant’s sex, age, weight, and height will be recorded, and they will complete a short survey about their eating and physical activity habits. Each participant will also be given advice about healthy lifestyle habits.

In no case will any names or identifying data be recorded.

If you want your child to participate in this work, please sign this letter and return it to your participating centre.

Yours sincerely,

Cristina Aparicio

SEFAC

Director of the Coordinating Centre

------------------------------------------------------

Student: _______________________________________

Course: ______________

Parent/guardian: _____________________________________________

Date: ___________________ Signature:

**ANNEX 2. Physical Activity Questionnaire for Adolescents (PAQ-A) in the 7 days prior.**

1. Physical activity in your spare time: Have you done any of the following activities in the past 7 days (last week)? If yes, how many times? *(Mark only one circle per row).*

|  | **No** | **1-2** | **3–4** | **5–6** | **7 times or more** |
| --- | --- | --- | --- | --- | --- |
| Skipping…………………………………………………… | O | O | O | O | O |
| In-line skating/rollerblading……………………………… | O | O | O | O | O |
| Tag………………………………………………………… | O | O | O | O | O |
| Bicycling…………………………………………………… | O | O | O | O | O |
| Walking (as an exercise) ………………………………… | O | O | O | O | O |
| Jogging or running………………………………………… | O | O | O | O | O |
| Aerobics/spinning ………………………………………… | O | O | O | O | O |
| Swimming ………………………………………………… | O | O | O | O | O |
| Dance……………………………………………………… | O | O | O | O | O |
| Badminton………………………………………………… | O | O | O | O | O |
| Rugby……………………………………………………… | O | O | O | O | O |
| Skateboarding …………………………………………… | O | O | O | O | O |
| Football/soccer/5-aside…………………………………… | O | O | O | O | O |
| Volleyball…………………………………………………… | O | O | O | O | O |
| Hockey……………………………………………………… | O | O | O | O | O |
| Basketball………………………………………………… | O | O | O | O | O |
| Ice skating/skiing………………………………………… | O | O | O | O | O |
| Other racket sports ……………………………………… | O | O | O | O | O |
| Handball…………………………………………………… | O | O | O | O | O |
| Athletics…………………………………………………… | O | O | O | O | O |
| Bodybuilding/weight-lifting ……………………………… | O | O | O | O | O |
| Martial Arts (e.g., Judo, Karate, etc.) …………………… | O | O | O | O | O |
| Others:______________________________________ | O | O | O | O | O |
| Others:______________________________________ | O | O | O | O | O |

2. In the last 7 days, during your physical education (PE) classes, how often were you very active (playing hard, running, jumping, throwing)? *(Check one only).*

| I don’t do PE …………………………… | O |
| --- | --- |
| Hardly ever……………………………… | O |
| Sometimes……………………………… | O |
| Quite often……………………………… | O |
| Always…………………………………… | O |

3. In the last 7 days, what did you normally do *at lunch* (besides eating lunch)? *(Check one only).*

| Sat down (talking, reading, doing schoolwork)……………… |
| --- |
| Stood around or walked around……………………………… |
| Ran or played a little bit ……………………………………… |
| Ran around and played quite a bit…………………………… |
| Ran and played hard most of the time………………………… |

4. In the last 7 days, on how many days *right after school* (until 6 p.m.), did you do sports, dance, or play games in which you were very active? *(Check one only).*

| None……………………………………… | O |
| --- | --- |
| 1 time last week………………………… | O |
| 2 or 3 times last week…………………… | O |
| 4 times last week……………………… | O |
| 5 times last week……………………… | O |

5. In the last 7 days, on how many *evenings* (between 6 p.m. and 10 p.m.) did you do sports, dance, or play games in which you were very active? *(Check one only)*.

| None……………………………………… | O |
| --- | --- |
| 1 time last week………………………… | O |
| 2 or 3 times last week…………………… | O |
| 4 times last week……………………… | O |
| 5 times last week……………………… | O |

6. *On the last weekend*, how many times did you do sports, dance, or play games in which you were very active? *(Check one only).*

| None……………………………………… | O |
| --- | --- |
| 1 time…………………………………… | O |
| 2 or 3 times……………………………… | O |
| 4 or 5 times……………………………… | O |
| 6 times…………………………………… | O |

7. Which *one* of the following describes you best for the last 7 days? Read *all five* statements before deciding on the *one* answer that describes you. *(Check one only).*

| All or most of my free time was spent doing things that involve little physical effort…………… | O |
| --- | --- |
| I sometimes (1–2 times last week) did physical things in my free time (e.g. played sports, went running, swimming, bike riding, did aerobics)… …………………………………………… | O |
| I often (3–4 times last week) did physical things in my free time………………………………… | O |
| I quite often (5–6 times last week) did physical things in my free time………………………… | O |
| I very often (7 or more times last week) did physical things in my free time…………………… | O |

8. Mark how often you did physical activity (like playing sports, games, doing dance, or any other physical activity) for each day last week.

|  | **None** | **Little bit** | **Medium** | **Often** | **Very often** |
| --- | --- | --- | --- | --- | --- |
| Monday………… | O | O | O | O | O |
| Tuesday………… | O | O | O | O | O |
| Wednesday…… | O | O | O | O | O |
| Thursday……… | O | O | O | O | O |
| Friday…………… | O | O | O | O | O |
| Saturday……… | O | O | O | O | O |
| Sunday………… | O | O | O | O | O |

9. Were you sick last week, or did anything prevent you from doing your normal physical activities? *(Check one).*

| Yes…………… | O |
| --- | --- |
| No…………… | O |

**ANNEX 3. Mediterranean Diet Quality Index in children and adolescents (KIDMED index)**

|  | YES | NO |
| --- | --- | --- |
| 1. Eat fruit or drink fruit juice daily |  |  |
| 1. Eat a second serving of fruit daily |  |  |
| 1. Eat fresh or cooked vegetables daily |  |  |
| 1. Eat fresh or cooked vegetables more than once daily |  |  |
| 1. Regularly eat fish (at least 2–3 time a week) |  |  |
| 1. Eat fast-food (e.g. a hamburger) in a restaurant once or more a week |  |  |
| 1. Eat legumes or pulses more than once a week |  |  |
| 1. Eat pasta or rice almost daily (5 or more times a week) |  |  |
| 1. Eat cereal or a cereal product for breakfast (bread, toast, etc.) |  |  |
| 1. Regularly eat nuts (at least 2 or 3 times a week) |  |  |
| 1. Use olive oil to cook at home |  |  |
| 1. Have breakfast every day |  |  |
| 1. Eat a dairy product for breakfast (milk, yogurt, etc.) |  |  |
| 1. Eat commercially baked goods or pastries for breakfast |  |  |
| 1. Eat two yogurts and/or cheese (40 g) every day |  |  |
| 1. Eat sweets or candy several times a day |  |  |
